# Immune Inflammation and Disease Progression in Idiopathic Pulmonary Fibrosis

**DOI:** 10.1371/journal.pone.0154516

**Published:** 2016-05-09

**Authors:** Elisabetta Balestro, Fiorella Calabrese, Graziella Turato, Francesca Lunardi, Erica Bazzan, Giuseppe Marulli, Davide Biondini, Emanuela Rossi, Alessandro Sanduzzi, Federico Rea, Chiara Rigobello, Dario Gregori, Simonetta Baraldo, Paolo Spagnolo, Manuel G. Cosio, Marina Saetta

**Affiliations:** 1 Department of Cardiac, Thoracic and Vascular Sciences, University of Padova, Padova, Italy; 2 Department of Clinical Medicine and Surgery, Federico II University, Napoli, Italy; 3 Respiratory Division Meakins-Christie Laboratories, McGill University, Montreal, Canada; Helmholtz Zentrum München, GERMANY

## Abstract

The clinical course in idiopathic pulmonary fibrosis (IPF) is highly heterogeneous, with some patients having a slow progression and others an accelerated clinical and functional decline. This study aims to clinically characterize the type of progression in IPF and to investigate the pathological basis that might account for the observed differences in disease behavior. Clinical and functional data were analyzed in 73 IPF patients, followed long-time as candidates for lung transplantation. The forced vital capacity (FVC) change/year (< or ≥10% predicted) was used to define “slow” or “rapid” disease progression. Pathological abnormalities were quantified in the explanted lung of 41 out of 73 patients undergoing lung transplantation. At diagnosis, slow progressors (n = 48) showed longer duration of symptoms and lower FVC than rapid progressors (n = 25). Eleven slow and 3 rapid progressors developed an acute exacerbation (AE) during follow-up. Quantitative lung pathology showed a severe innate and adaptive inflammatory infiltrate in rapid progressors, markedly increased compared to slow progressors and similar to that observed in patients experiencing AE. The extent of inflammation was correlated with the yearly FVC decline (r = 0.52, p = 0.005). In conclusion an innate and adaptive inflammation appears to be a prominent feature in the lung of patients with IPF and could contribute to determining of the rate of disease progression.

## Introduction

Idiopathic pulmonary fibrosis (IPF) is a specific form of chronic progressive fibrosing interstitial lung disease of unknown cause that carries a dismal prognosis, with a 5-years survival of approximately 20% [1].

A chronic inflammatory process of the lung has long been considered the main mechanism underlying IPF [2–6]. However, recently there has been a conceptual transition in IPF pathogenesis from an inflammatory driven process to a primarily fibrotic one, in which fibrogenesis results from recurrent microinjury of alveolar epithelial cells followed by aberrant repair processes leading to fibrosis [7–9]. Currently the inflammatory process is described as mild and consisting of a patchy interstitial infiltrate of lymphocytes and plasma cells [10, 11] and is not considered an important component of IPF pathology or a factor contributing to pathogenesis of the disease.

The clinical course of IPF is highly heterogeneous, with the majority of patients having a relatively slow progression while the remainder have an accelerated, rapid decay in lung function and shorter survival [12–14]. In this regard, it has been reported that the global gene expression in the rapid and slow progression phenotypes differs significantly, with up-regulation of molecular pathways involved in cell motility, fibroblast differentiation and inflammation in the rapid progressors [13, 14]. Further evidence that IPF does not behave clinically as a single entity stems from the observed differences in the expression of inflammatory markers and autoantibodies that are associated with different clinical outcomes in patients with IPF [15–23]. Furthermore, patients with IPF can develop an acute exacerbation (AE) of their disease and this carries a poor prognosis [24]. In spite of this heterogeneous clinical behaviour, these observations have not been incorporated in the practical approach to the disease, perhaps because there is insufficient information about the clinical course and corresponding lung pathology in these patients to justify the segregation of these groups.

In this study we aimed to characterize the type of disease progression, slow or rapid, in a group of patients with IPF followed for long time before undergoing lung transplantation, and correlate it with a detailed quantitative study of the pathology of the explanted lung. Indeed we hypothesized that the different clinical behaviour may be accounted for, at least partially, by the different lung pathologies. This information might potentially add significantly to the understanding of pathogenesis and disease behaviour of IPF.

## Methods

In this study, we defined the clinical and functional progression in a group of 73 IPF patients referred for possible lung transplantation to our center in Padova between 2000 and 2014. In the 41 patients from this group who underwent lung transplantation we performed a quantitative pathological study of the native lung and compared the findings in lung pathology with the clinical progression.

### Clinical analysis

Seventy three patients with IPF referred to our center for possible lung transplantation were included in the study. IPF was diagnosed according to the ATS/ERS or the ATS/ERS/JRS/ALAT Guidelines (according to whether they were referred before or after the publication of the 2011 guidelines) [1, 10, 11]. Information collected retrospectively was integrated with a longitudinal follow-up to determine the clinical progression of IPF (slow or rapid) from the beginning of symptoms to lung transplantation, death or end of follow-up (up to December 2014).

Medical records were reviewed and data on serial lung function tests, high-resolution computed tomography (HRCT) of the chest, right heart catheterization and/or echocardiography were collected for all patients. None of the subjects had a clear history of occupational or environmental exposure to fibrogenic agents, nor clinical features of hypersensitivity pneumonitis or connective tissue disease. All patients had negative autoimmune serologic testing including antinuclear antibodies (ANA), anti-double strand (anti-ds) DNA antibodies, anti-extractable nuclear antigens (ENA), antineutrophil cytoplasm antibodies (ANCAs) and cyclic citrullinated peptide (CCP). The study population included 2 brothers with familial IPF.

During follow-up, spirometry was serially performed every 6 to 12 months. The fall in % predicted FVC per year was used to characterize the disease progression as “rapid” (fall in % predicted FVC ≥ 10% per year) and “slow” (fall in % predicted FVC < 10% per year) as previously reported [14] (for details, see Clinical analysis in S1 File).

The study was performed according to the Declaration of Helsinki and was approved by the Ethics Committee for Clinical Experimentation of Padova. Written consent was obtained from all subjects. None of the transplant donors were from a vulnerable population and all donors or next of kin provided written informed consent that was freely given.

### Pathological analysis

Forty one of the 73 patients underwent lung transplantation. The native lungs were fixed in formalin by airway perfusion and samples from upper and lower lobes were obtained and embedded in paraffin. Sections 5 μm-thick were cut and stained for histological and immunohistochemical analysis.

In all transplanted cases the diagnosis of IPF was confirmed by our expert pathologist by the presence of an usual interstitial pneumonia (UIP) pattern [10, 11]. Fibroblastic foci and lymphoid follicles were counted in sections stained with hematoxylin–eosin and antiCD20 respectively and expressed as number per square cm of area examined. Diffuse alveolar damage (DAD), defined as stratified hyaline membranes, was assessed on sections stained with haematoxylin-eosin and considered as “present” when detected in at least 30% of each lung section. Results were expressed as % of patients with presence of DAD over total number of patients.

Cellular inflammatory infiltrate comprising total leukocytes (CD45^+^), neutrophils, macrophages (CD68^+^), CD4^+^ and CD8^+^ T lymphocytes as well as B lymphocytes (CD20^+^) was identified by immunohistochemistry as previously described [25–26]. Each inflammatory cell type was quantified in 20 non overlapping high power fields per slide and expressed as cells/mm^2^ of area examined. Morphometric analysis was performed by an experienced researcher (GT) and the intraobserver and interobserver reproducibility were than assessed (for details, see Pathological analysis in S1 File). A representative figure of each inflammatory cell type is reported in Figures A-E in S1 File.

### Statistical analysis

Differences between groups were analyzed using Mann-Whitney U test and Fisher Exact test, as appropriate. The relationship between different outcomes was evaluated using Spearman’s rank correlation. Multivariate regression analysis was performed to investigate if any of the clinical data available at diagnosis was associated with the rapid or slow rate of decline.

## Results

### Clinical analysis

**Fig 1:**
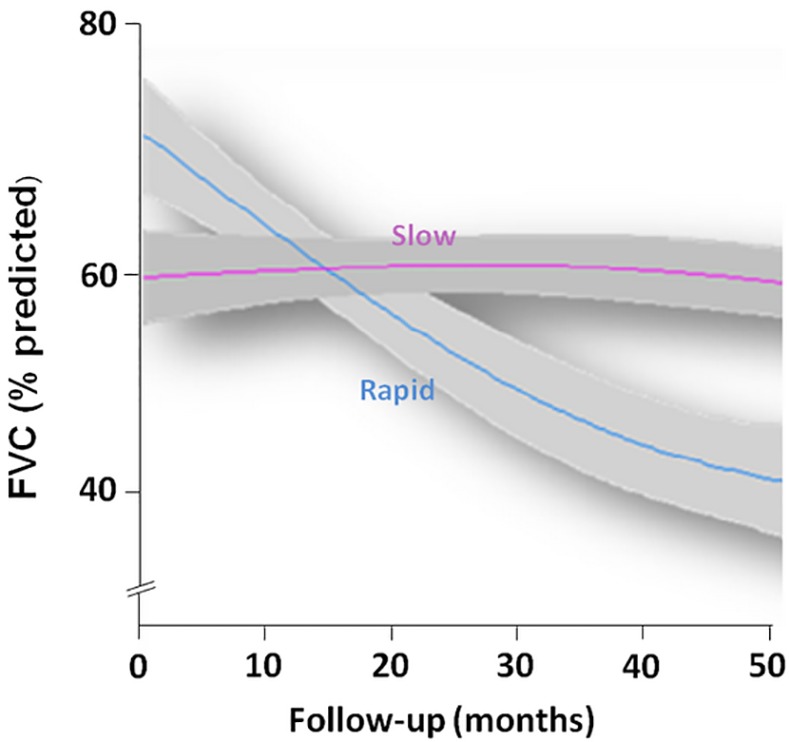
Rate of FVC % predicted decline over time. Time course of the % predicted FVC change in slow and rapid decliners obtained using all the FVC measurements performed during follow-up for each patient. Trends have been estimated using a linear model allowing for non-linearity of time trends, estimated via cubic-splines. The longitudinal trends of the slow and rapid declines are significantly different (p <0.001). The % predicted FVC at diagnosis (time 0) is significantly higher in rapid than in slow decliners (see Table 1).

Forty-eight of the 73 cases (66%) had a slow FVC decline while 25 (34%) had a rapid decline. Their time course decline during the follow-up period is shown in Fig 1. Demographics and clinical characteristics of slow and rapid progressors are summarized in Table 1.

**Table 1:** Clinical characteristics of slow and rapid progressors.

	Slow (n = 48, 66%)	Rapid (n = 25, 34%)	p value
**Age at diagnosis–years**	**54(36–64)**	**54(33–69)**	
**Male sex–n. %**	**37(77%)**	**20(80%)**	
**Smokers–n. %**	**35(73%)**	**18(72%)**	
**Smoking history (only smokers)–pack-years**	**25(0.1–120)**	**24(3–93)**	
**Duration of symptom before diagnosis–months**	**31(2–96)**	**6(1–58)**	**0.0031**
**FVC at diagnosis–% predicted**	**61(20–94)**	**70(46–108)**	**0.022**
**FEV**_**1**_ **at diagnosis—%predicted**	**64(26–97)**	**69(45–118)**	
**FEV**_**1**_**/FVC at diagnosis—%**	**84(71–111)**	**80(73–89)**	
**DLco at diagnosis–% predicted**	**40 (3–100)**	**38(10–85)**	
**FVC at end follow up–% predicted**	**52(21–87)**	**38(22–91)**	**0.0025**
Follow up (from diagnosis to end study*)–months	**36(7–158)**	**24(12–60)**	**0.015**
**6MW distance–mt**	**270(40–437)**	**230(48–390)**	
**mPAP–mmHg**	**20(10–51)**	**20(13–45)**	
**FVC decline/year -% predicted**	**2.8(0.0–9.1)**	**14.2(10.2–28.0)**	**<0.0001**
**FVC decline/year—ml**	**98(0–416)**	**480(297–1499)**	**<0.0001**
**AE–n %**	**11(23%)**	**3(12%)**	

Values are expressed as numbers and (%) or medians and (ranges)

* transplant (n = 41), death (n = 14) or end follow-up (n = 18)

Age, sex and smoking habits were similar in the two groups. The duration of symptoms before diagnosis was significantly shorter in rapid as compared to slow progressors. At diagnosis, functional characteristics, including diffusing capacity of the lung for carbon monoxide (DLco) and FEV_1_, were similar in the two groups. However, against expectations, the percent predicted FVC at diagnosis was significantly lower in slow than in rapid progressors. By contrast, and as expected, at the end of follow-up the percent-predicted FVC was significantly lower in rapid compared to slow progressors (Table 1). Six minute walking test distance and pulmonary arterial pressure as assessed by either transthoracic echocardiography or right heart catheterization at the time of consideration for transplantation, were similar in slow and rapid progressors (Table 1). As expected by the group definition, the annual decline in percent predicted FVC was lower in slow than in rapid progressors. This difference in functional decline was even more pronounced when the FVC fall was expressed in absolute terms as ml/year (98 ml/year *vs* 480 ml/year in slow and rapid progressors respectively) (Table 1).

Multivariable analysis showed that a lower FVC at diagnosis (OR: 0.22, 95% CI: 0.06–0.71, p = 0.012) and a long duration of symptoms before diagnosis (OR: 6.56, 95% CI: 1.66–25.59 p = 0.023) were the only clinical variables associated with a slow FVC decline during the follow up. The combination of these two variables had an additive predictive effect.

During the follow up, 14 of the 73 patients (19%) developed an acute exacerbation (AE) defined according to the diagnostic criteria proposed by Collard et al [24] (see Clinical analysis in S1 File). Notably, most of the AE (11 of 14) occurred in the slow progression group.

Patients were treated with different regimens based on prednisone with or without azathioprine according to existing guidelines. There were no differences in treatment regimens between slow and rapid progressors.

### Pathological analysis

**Fig 2:**
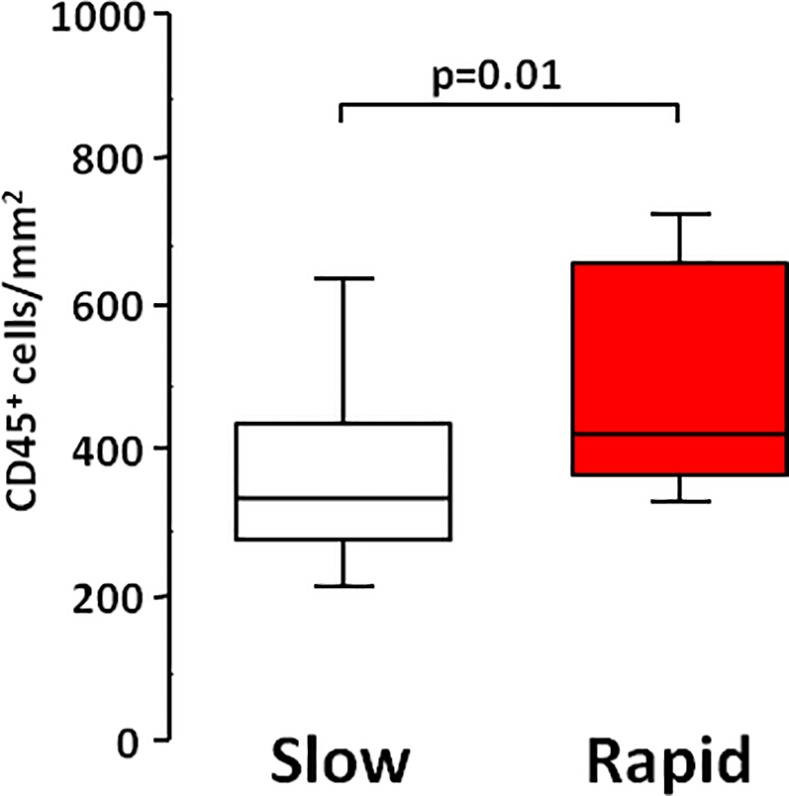
Total inflammatory cells in slow and rapid progressors. Number of total leukocytes (CD45^+^/mm^2^) in the lungs of slow and rapid progressors. Horizontal bars represent median values; bottom and top of each box plot 25^th^ and 75^th^, brackets 10^th^ and 90^th^ percentiles. Slow: white; rapid: red.

**Fig 3:**
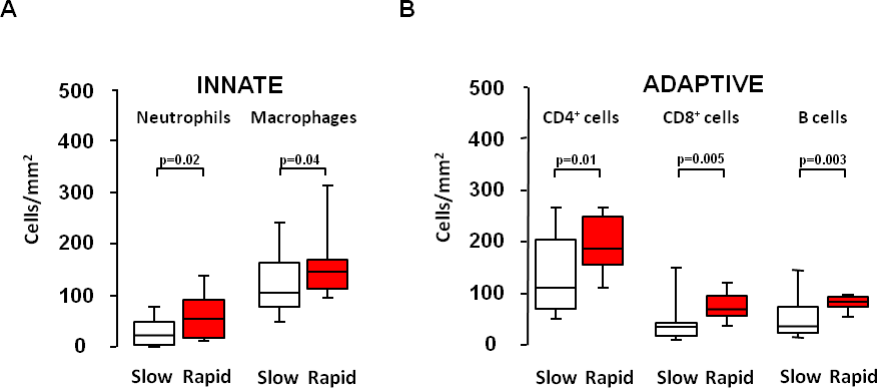
Differential Inflammatory cells in slow and rapid progressors. Number of innate inflammatory cells (neutrophils and macrophages) and adaptive inflammatory cells (CD4^+^, CD8^+^, and B lymphocyte) in the lungs of slow and rapid progressors. Horizontal bars represent median values, bottom and top of each box plot 25^th^ and 75^th^, brackets 10^th^ and 90^th^ percentiles. Slow: white; rapid: red.

Lung pathology was examined in those patients who underwent lung transplantation (n = 41): 27 slow progressors and 14 rapid progressors. The clinical characteristics of these 41 transplanted patients (Table B in S1 File) were comparable to the 73 patients in the whole group. Morphometric analysis of the explanted lung showed a prominent cellular inflammatory infiltrate with the number of total leukocytes/mm^2^ (CD45^+^) being significantly higher in rapid than in slow progressors (p = 0.01) (Fig 2). Both innate (neutrophils and macrophages) and adaptive (CD4^+^, CD8^+^ and B lymphocytes) inflammatory cell numbers were significantly higher in the rapid progressor group compared to the slow one (Fig 3).

**Fig 4:**
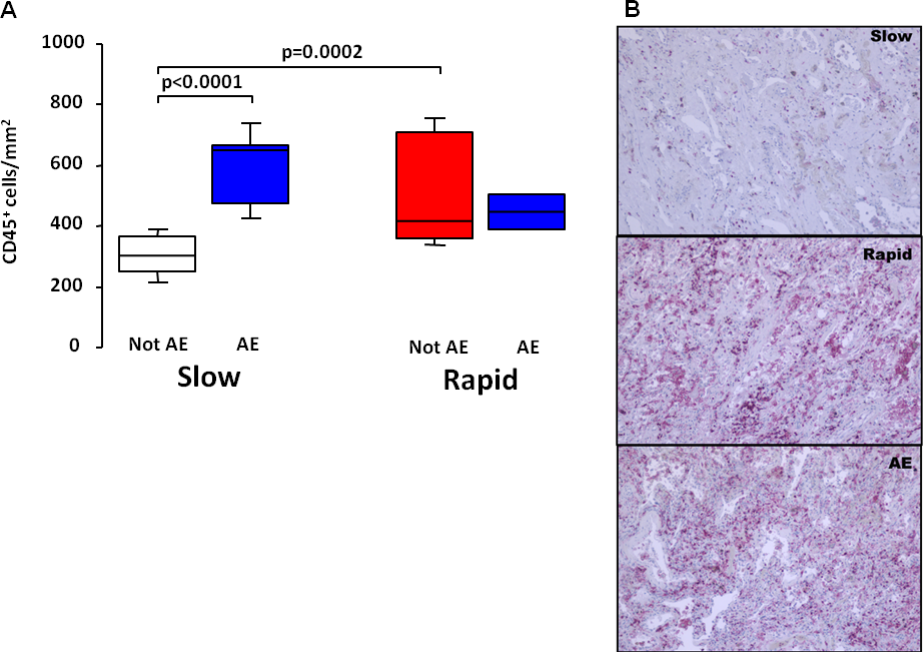
Total inflammatory cells in slow and rapid progressors with and without acute exacerbation (AE). A. Numbers of total leukocytes (CD45^+^/mm^2^) in the lungs of slow and rapid progressors; subjects who developed and those who did not develop AE in each group are considered separately. Horizontal bars represent median values, bottom and top of each box plot 25^th^ and 75^th^, brackets 10^th^ and 90^th^ percentiles. Slow not AE: white; rapid not AE: red; AE: blue. B. Microphotographs showing total leukocyte (CD45^+^) infiltration (in red) in the lung of a slow decliner, a rapid decliner and an AE patient. Original magnification X10; immunostaining with anti-CD45.

**Fig 5:**
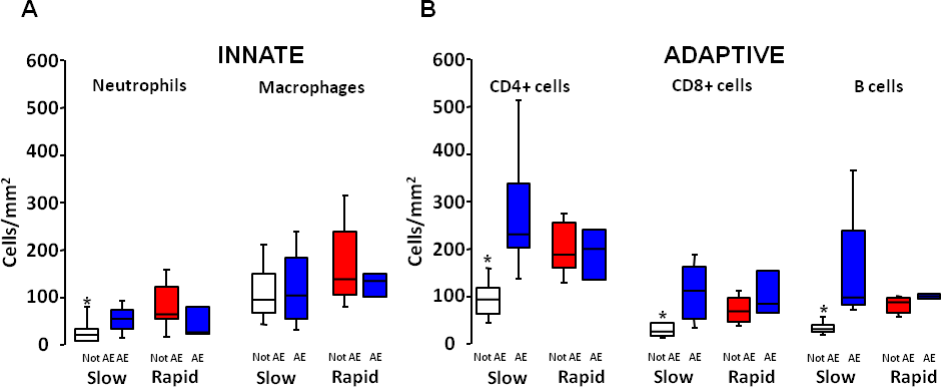
Differential inflammatory cells in slow and rapid progressors with and without acute exacerbation (AE). Number of innate inflammatory cells (neutrophils, macrophages) and adaptive inflammatory cells (CD4^+^, CD8^+^, and B lymphocytes) in the lungs of slow and rapid progressors; subjects who developed and those who did not develop AE are considered separately. Horizontal bars represent median values, bottom and top of each box plot 25^th^ and 75^th^, brackets 10^th^ and 90^th^ percentiles. Slow not AE: white; rapid not AE: red; AE: blue. * P<0.001 compared to slow who develop AE and rapid who do not develop AE

Eleven of the 14 patients who develop AE during the follow-up period underwent lung transplantation (8 slow and 3 rapid progressors). In view of the relevant number of subjects who developed an AE and in order to better understand their underlying pathology, we analyzed separately the subjects who developed and those who did not develop AE in both slow and rapid progressors (the clinical characteristics of the 4 groups are reported in the Table C in S1 File). The slow progressors who developed AE showed a more marked overall inflammation (CD45^+^) than the slow progressors who did not develop AE (Fig 4A). No difference was observed in the rapid progressors where a severe inflammation was present in both those with AE and those without AE. Indeed, the degree of inflammation in the rapid progressors who did not exacerbate was similar to that in the acute exacerbators, irrespective of whether they were originally slow or rapid progressors (Fig 4A and 4B). The immune inflammatory infiltrate comprised both innate (neutrophils) and adaptive (CD4^+^, CD8^+^ and B lymphocytes) cells, all of which were found in significantly higher numbers in rapid progressors and acute exacerbators than in slow progressors without AE (Fig 5). Of interest diffuse alveolar damage (DAD), that is considered a characteristic feature of AE, was also found to the same extent in rapid progressors who did not exacerbate. No difference was found in the numbers of fibroblastic foci and lymphoid follicles among the four groups of subjects (Table 2), however the number of lymphoid follicles in IPF lungs overall was very high, more than what is usually seen in COPD [27] where lymphoid follicles are a prominent feature. Of interest, in our study population, emphysema was neither seen radiologically nor pathologically.

**Table 2:** Pathological characteristics of slow and rapid progressors with and without AE.

	Slow (n = 27)		Rapid (n = 14)	
	Not AE (n = 19)	AE (n = 8)	Not AE (n = 11)	AE (n = 3)
**DAD**	4 (21%)*	**6 (75%)**	**10 (91%)**	**3 (100%)**
**Fibroblastic Foci—n/cm**^**2**^	**4.7 (0.4–11.7)**	**2.2 (1.9–4.1)**	**3 (1–7.3)**	**4 (1.1–6.1)**
**Lymphoid follicles—n/cm**^**2**^	**5(1–16)**	**9(2–16)**	**6(2–11)**	**9(9–11)**

DAD is expressed as number (%) of subjects with presence of DAD in their lung sections.

Fibroblastic foci and lymphoid follicles are expressed as medians and (ranges).

* p<0.05 compared to AE

No differences were found in the clinical features, including the rate of progression and number or type of inflammatory cells, between smokers and non-smokers in any of the groups examined (Tables D-F in S1 File).

In slow and rapid progressors who did not develop AE the number of total leukocytes (CD45^+^) was positively correlated to the yearly decline in FVC (r = 0.52, p = 0.005) and showed a negative trend with the duration of symptoms before diagnosis (r = -0.35, p = 0.05).

## Discussion

In the present study we describe the clinical course of a group of patients with IPF referred to our center for lung transplantation and characterize their pathological features. We found that two clinical phenotypes can be clearly identified (slow and a rapid progressors) and that substantial differences in their pathological features are present, with the degree of inflammation, involving innate and adaptive immunity, being the most striking one. Conceivably these differences in lung pathology might be important contributors to the different clinical behaviour.

Using the criteria proposed by Boon and colleagues [14], based on the fall in percent predicted FVC/year, we differentiated our patients into slow (66%) and rapid (34%) progressors. On average, the absolute yearly fall in FVC was 98 ml/year in the “slow” decliners and 480 ml/year in the “rapid” ones. The median fall for the whole population was 215 ml/year, a value similar to that recently reported in large clinical trials [28, 29], which suggests that our population is well representative of the IPF population in general. In view of the clinical importance of the different progression, we investigated whether the rate of decline could be predicted at diagnosis based on any of the clinical and/or functional variables available at presentation. We found that a low value of FVC at diagnosis and a long duration of symptoms before diagnosis, as previously described by Selman and colleagues [13], were the best fitting predictors for the likelihood of having a slow, instead of a rapid, FVC decline. The possibility of distinguishing between slow and rapid progressors early in the disease course is clinically relevant, especially in view of the current availability of medical therapies that are effective in slowing functional decline and disease progression in the early stage of the disease [28–31].

It is of interest that, at presentation, rapid progressors had a higher % predicted FVC than the slow progressors, a finding somehow unexpected. A possible explanation is that a more rapid change in FVC in the rapid decliners is perceived more readily, thus promoting a faster seek for medical attention. This finding is important since it may suggest that the value of the FVC at diagnosis by itself does not reflect the pre-diagnosis rapidity of decline nor can predict the future rate of progression [32], unless the duration of symptoms before diagnosis is also taken in consideration, as our data suggest.

Acute exacerbation (AE), an acute worsening of the underlying disease without obvious cause [24], is a clinically relevant and often fatal event in IPF. The incidence of AE estimated from clinical trials with short follow-up is between 5 and 10% [1, 24]. However, 19% of our patients developed AE during follow-up and the majority of them, 11 out of 14, belonged to the slow declining group, in line with previous reports [13]. The high rate of AE in our study might be due to the long follow up period or, more likely, to the fact that being a transplant centre, we followed a selected population. The high incidence of AE seen in our population is in line with a recently reported incidence of 20.7% in a 3-years follow up study in IPF [33].

Once we had defined the different clinical course in our population, we hypothesized that different progression might be associated with distinct underlying pathological findings. Indeed, important differences in lung pathology were observed between slow and rapid progressors, consisting mainly on the presence of an extensive degree of innate and adaptive immune inflammation in the rapid group, more prominent than in the slow progressors. Of interest, we did not observe differences in lung pathology between subjects who experienced an AE and rapid progressors who did not exacerbate, including the presence of DAD, an abnormality considered a characteristic feature of AE. This observation would support the hypothesis that rapid progression might be the consequence of repeated, but silent, acute events. The relation between the occurrence of AE and the type of clinical progression is of interest. We have found in our population that the majority of AE occurred among the slow progressors, and that the main difference between patients with an AE and slow progressors who did not develop an exacerbation, is the extent of lung immune inflammation. Therefore, it could be speculated that an idiopathic or induced “acceleration” of the generally “contained” immune inflammatory reaction present in slow progressors could account for the exuberant clinical and pathological picture seen in AE.

The results of the detailed quantitative pathological analysis in IPF lungs appear to support a contribution of inflammation in determining disease behaviour in IPF. Despite the increased evidences that immunology might play a role in IPF [15–23, 34–40], inflammation is not considered an important component of UIP or a factor contributing to the progression or pathogenesis of the disease. Furthermore, previous pathological descriptions, performed in lung biopsies from IPF patients at the time of diagnosis, reported no differences in pathology between the slow and rapid decliners [13]. Unfortunately, we could not examine the lung pathology at the time of diagnosis in our patients, so we do not know if some differences were already present at that stage.

The presence of an exuberant immune inflammatory infiltrate, found predominantly in the rapid progressors, is consistent with the gene expression profile reported by Boon and colleagues [14], who indeed described the activation of important pro-inflammatory pathways that may potentially play a role in the immune activation and disease progression of the rapid decliners [14]. Our findings are also in keeping with previous observations showing that lymphocyte density in IPF lung is associated with FVC decline [21] and poor survival [34]. Moreover, in keeping with an important role of immune activation and inflammation, recent publications suggest that increased expression of pro-inflammatory factors may predispose to worse outcomes in IPF [15–23, 35–38]. For instance, CXCL13, a chemokine involved in B cell trafficking and recruitment, has been shown to be related with disease severity and survival [20, 37]. In line with these reports is also the presence of B cell aggregates [20, 39, 40] and highly differentiated circulating B cells in patients with IPF [39], findings usually observed in autoimmune syndromes. In this context, it is of interest to note that autoantibodies to heat shock protein 70 [15] and periplakin [23], which have been identified in serum and bronchoalveolar lavage of patients with IPF, have been reported to be associated with a more severe disease, further supporting the presence of an autoimmune response in the pathogenesis of IPF. In this regard our finding of an increased number of B lymphocytes in the lungs of rapid progressors supports a potential role for these cells, along with T lymphocytes, in the development of an adaptive immune response, possibly contributing to the course of the disease.

Of interest the two drugs recently reported to be effective in the treatment of IPF, Nintedanib and Pirfenidone [28, 29], beside their antifibrotic effect are known to have even antinflammatory properties, hence some of their action could be mediated by a control of the immune inflammation present in IPF lungs.

We are well aware that by studying the pathology of the explanted lung we are looking at the “terminal”, or at least advanced, stages of the disease. In other words, we may be looking at the consequence rather than the cause of the pathologic process. Yet, the clear differences in pathology found between slow and rapid progressors, both at a terminal stage of their disease, validate the significance of our findings and indicate that the “end stage” itself cannot explain the enhanced immune inflammatory process observed in rapid progressors.

Likely our findings in explanted lungs showing the possible role of inflammation in the different patterns of progression, slow and rapid, may help to understand better the disease. Furthermore, this knowledge might allow the search of other ways of assessing inflammatory and immune mechanisms, without biopsies, either by blood and bronchoalveolar lavage (BAL) analysis or by radiology.

In conclusion, in a cohort of IPF patients referred for lung transplantation, an innate and adaptive inflammatory process of the lung is a significant feature and appears to be an important determinant of the rate of disease progression. These findings may provide a basis for future investigation on predicting outcomes and personalized treatment in this heterogeneous disease.

## Supporting Information

S1 File Additional Details. (DOCX)

## Abbreviations

AE: Acute Exacerbation
DAD: Diffuse Alveolar Damage
FEV_1_: Forced Expiratory Volume in the 1^st^ second
FVC: Forced Vital Capacity
IPF: Idiopathic Pulmonary Fibrosis
