# Supplementary material for: Immune Inflammation and Disease Progression in Idiopathic Pulmonary Fibrosis
Source: PLoS One. 2016 May 9;11(5):e0154516. doi: 10.1371/journal.pone.0154516 (PMC4861274; doi:10.1371/journal.pone.0154516)
Supplement: S1 File — (DOCX) [file pone.0154516.s001.docx]

**Supporting Information File S1**

**Additional Details**

**Immune inflammation and disease progression in idiopathic pulmonary fibrosis**

**Elisabetta Balestro, Fiorella Calabrese, Graziella Turato, Francesca Lunardi, Erica Bazzan, Giuseppe Marulli, Davide Biondini, Emanuela Rossi, Alessandro Sanduzzi, Federico Rea, Chiara Rigobello, Dario Gregori, Simonetta Baraldo, Paolo Spagnolo, Manuel G Cosio, Marina Saetta**

**METHODS**

**Clinical analysis**

In each subject the fall in % predicted FVC per year was used to characterize the disease progression as “rapid” (fall in % predicted FVC ≥ 10% per year) and “slow” (fall in % predicted FVC < 10% per year) as previously reported (1). For this categorization, the difference between the FVC value at diagnosis and that at the end of the study (prior to transplant, death or end follow-up) was calculated, expressed as % predicted and normalized per year.

In 41 subjects in whom the FVC value 12 months after diagnosis was available and follow-up was of at least 2 year (30 “slow” and 13 “rapid”), we showed that the change in FVC in the 1st year was representative of the rate of progression during the whole follow up in all subjects except 1.

During follow-up 14 out of the 73 patients developed an acute exacerbation (AE) defined by a combination of unexplained worsening of dyspnea within 1 month, evidence of hypoxemia as defined by worsened or severely impaired gas exchange, new radiographic alveolar infiltrates, and an absence of an alternative explanation such as infection, pulmonary embolism, pneumothorax, or heart failure (2)

**Pathological analysis**

Forty-one out of the 73 IPF patients underwent lung transplantation. The whole native lungs were gently fixed in 10% phosphate-buffered formalin by airway perfusion at constant pressure. Samples from both upper and lower lobes were taken and processed and 5 μm-thick sections were stained for histological and immunohistochemical analysis.

Interstitial inflammation was precisely characterized with immunohistochemical methods identifying total leukocytes (CD45^+^), neutrophil (elastase^+^), macrophages (CD68^+^), T lymphocytes (CD4^+^ and CD8^+^) and B lymphocyte (CD20^+^) as previously reported (3). Some details are reported in Table A.

Table A: Antibodies and detection systems

| Antibody | Dilution | Company and Catalogue number | Antigen retrieval method | Antigen detection |
| --- | --- | --- | --- | --- |
| Monoclonal mouse anti-CD45  (2B11 + PD7/26) | 1:60 | DAKO  M 0701 | Microwave in citrate buffer pH 6 | Anti-mouse immunoglobulins coniugated with alkaline phosphatase and liquid permanent red |
| Monoclonal mouse anti-CD4  (Clone 4B12) | 1:50 | DAKO  M 7310 | Microwave in EDTA buffer pH 8 | Envision detection system, peroxidase and diaminobenzidine (DAB) |
| Monoclonal mouse anti-CD8  (Clone C8/144B) | 1:50 | DAKO  M 7103 | Microwave in citrate buffer pH 6 | Anti-mouse immunoglobulins coniugated with alkaline phosphatase and liquid permanent red |
| Monoclonal mouse anti-CD20  (Clone L26) | 1:100 | DAKO  M 0755 | Microwave in citrate buffer pH 6 | Envision detection system, peroxidase and diaminobenzidine (DAB) |
| Monoclonal mouse anti-CD68  (Clone PG-M1) | 1:500 | DAKO  M 0876 | Microwave in citrate buffer pH 6 | Anti-mouse immunoglobulins coniugated with alkaline phosphatase and liquid permanent red |
| Monoclonal mouse human Neutrophil-elastase  (Clone NP57) | 1:150 | DAKO  M 0752 |  | Anti-mouse immunoglobulins coniugated with alkaline phosphatase and liquid permanent red |

Each inflammatory cell type was quantified in 20 non consecutive high power fields per slide. In particular, inflammatory cells were assessed trough the slide counting inflammatory cells in one microscopic high power field, overlooking the three consecutive fields and counting the fourth one. Results were expressed as number of positive cells per square mm of area examined. Morphometric analysis was performed by one observer with 20 years experience (G.T.). In a subgroup of patients, the same measurements were performed again by the same observer to assess the intraobserver reproducibility, and by a second observer (E.B.) to assess the interobserver reproducibility.

The intraobserver correlation coefficient for total inflammation was 0.96 while the interobserver correlation coefficient was 0.92. The average variability on scores between sections was 21% (CI 17%-25%).

A representative figure of each inflammatory cell type is reported below (Figures A-E).

To better characterize the inflammatory process, lymphoid follicles (aggregates containing more than 40 contiguous mononuclear cells that demonstrated the characteristic topographical arrangement of B cells (CD20^+^) and T cells) were counted in sections stained with anti-CD20 and expressed as number of lymphoid follicles per square cm of area examined, as previously reported (4).

Figure A

**
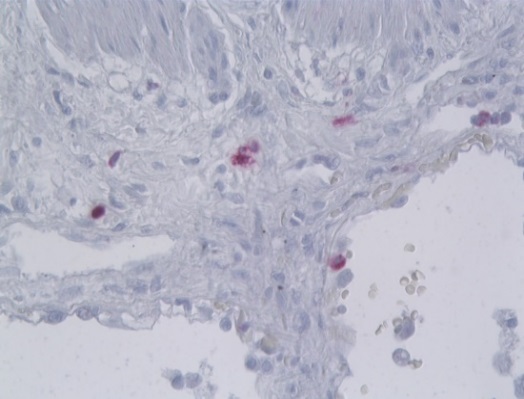

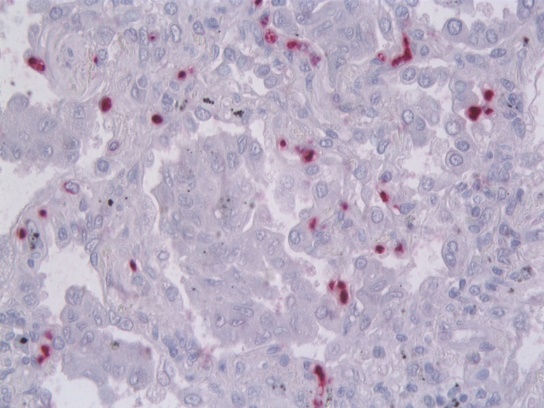
**

**b**

**a**

Microphotographs showing neutrophil infiltration (in red) in the lung of a slow decliner (a) and a rapid decliner (b). Original magnification X20; immunostaining with anti-neutrophils elastase.

Figure B


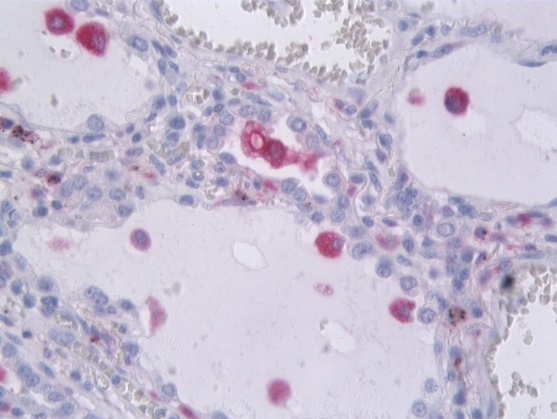

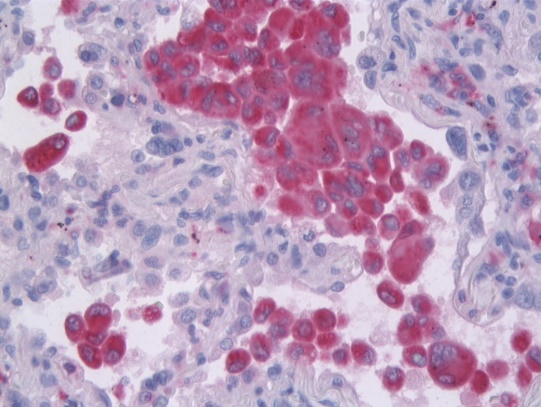


**b**

**a**

Microphotographs showing macrophage infiltration (in red) in the lung of a slow decliner (a) and a rapid decliner (b). Original magnification X20; immunostaining with anti-CD68.

Figure C

**
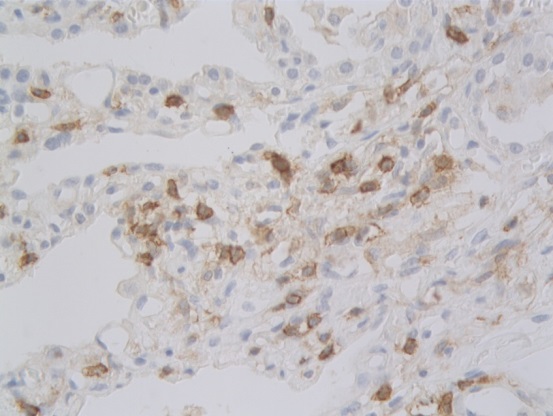

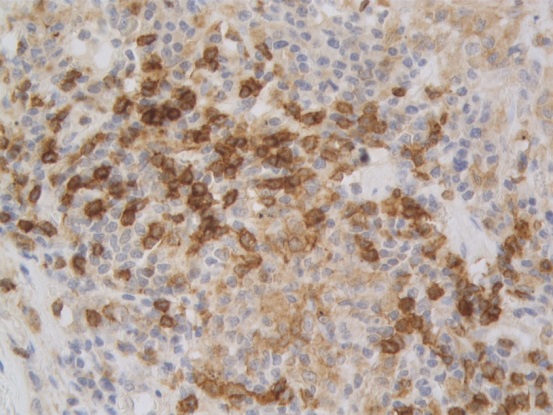
**

**b**

**a**

Microphotographs showing CD4^+^ cell infiltration (in brown) in the lung of a slow decliner (a) and a rapid decliner (b). Original magnification X20; immunostaining with anti-CD4.

Figure D

**
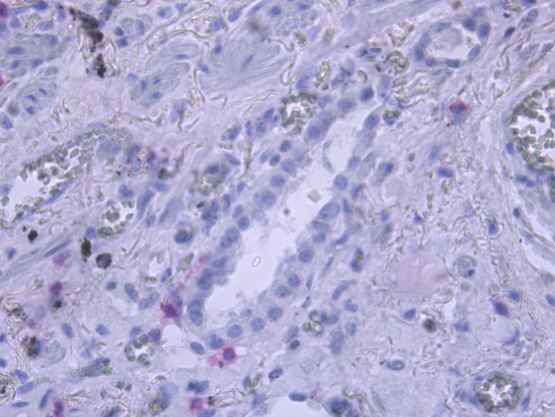

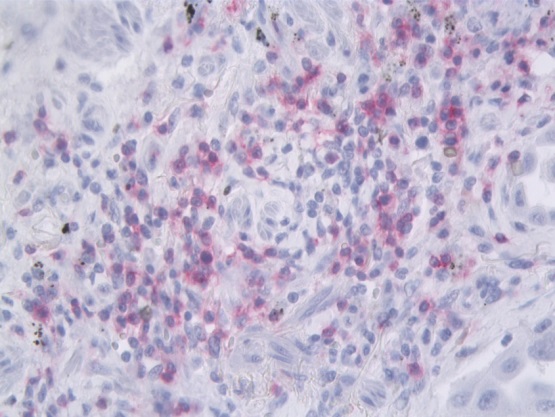
**

**b**

**a**

Microphotographs showing CD8^+^ T cell infiltration (in red) in the lung of a slow decliner (a) and a rapid decliner (b). Original magnification X20; immunostaining with anti-CD8.

Figure E

**
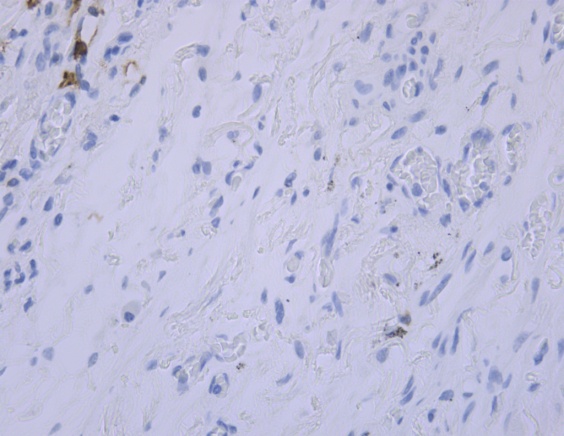

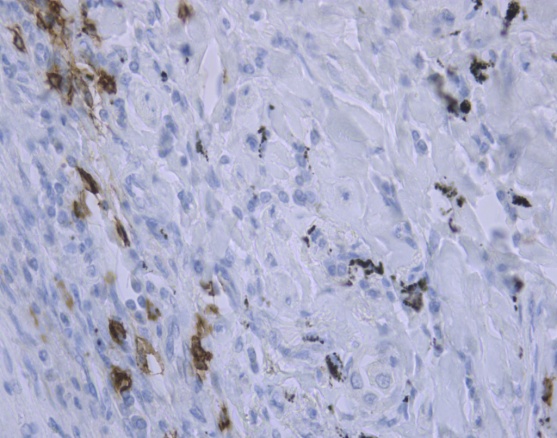
**

**b**

**a**

Microphotographs showing B lymphocyte infiltration (in brown) in the lung of a slow decliner (a) and a rapid decliner (b). Original magnification X20; immunostaining with anti-CD20.

**RESULTS**

**Table B. Clinical characteristics of slow and rapid progressors who underwent lung transplantation compared to the whole group**

|  | **Transplanted subjects**  **n=41** | | **All subjects**  **n=73** | |
| --- | --- | --- | --- | --- |
|  | **Slow**  **n=27(66%)** | **Rapid**  **n=14)** | **Slow**  **n=48 (66%)** | **Rapid**  **n=25 (34%)** |
| **Age at diagnosis – years** | **54(40-64)** | **53(33-64)** | **54(36-64)** | **54(33-69)** |
| **Male sex – n. %** | **20(74%)** | **12(86%)** | **37(77%)** | **20(80%)** |
| **Smokers – n. %** | **18(67%)** | **11(79%)** | **35(73%)** | **18(72%)** |
| **Smoking history – pack-years**  **Only smokers** | **29(1-120)** | **22(3-93)** | **25(0.1-120)** | **24(3-93)** |
| **Duration of symptom before diagnosis – months** | **36(2-75)**** | **6(1-44)** | **31(2-96)**** | **6(1-58)** |
| **FVC at diagnosis – % predicted** | **57(20-93)**** | **73(56-86)** | **61(20-94)*** | **70(46-108)** |
| **FEV_1_ at diagnosis - %predicted** | **54(25-93)**** | **70(56-85)** | **64(26-97)** | **69(45-118)** |
| **FEV_1_/FVC at diagnosis - %** | **82(71-111)** | **80(73-89)** | **84(71-111)** | **80(73-89)** |
| **DLco at diagnosis – % predicted** | **36(15-79)** | **34(10-85)** | **40 (3-100)** | **38(10-85)** |
| **FVC at end follow up –% predicted** | **45(21-87)** | **38(23-69)** | **52(21-87)**** | **38(22-91)** |
| **Follow up (from diagnosis to end study^#^) – months** | **41(7-72)** | **30(12-46)** | **36(7-158)*** | **24(12-60)** |
| **6MW distance – mt** | **270(40-437)** | **238(48-390)** | **270(40-437)** | **230(48-390)** |
| **mPAP – mmHg** | **20(10-51)** | **20(13-35)** | **20(10-51)** | **20(13-45)** |
| **FVC decline/year -%predicted** | **1.3(0.0-9.1)***** | **11.2(10.2-28.0)** | **2.8(0.0-9.1)***** | **14.2(10.2-28.0)** |
| **FVC decline/year - ml** | **50(0-339)***** | **467(340-1498)** | **98(0-416)***** | **480(297-1499)** |
| **AE – n %** | **8(29%)** | **3(21%)** | **11(23%)** | **3(12%)** |

Values are expressed as numbers and % or medians and (ranges)

^#^ transplant, death or end follow-up

*P<0.05; **P<0.01; ***P<0.0001 when slow were compared to rapid progressors

**Table C. Clinical characteristics of slow and rapid progressors with and without AE**

|  | **SLOW**  **n=48** | | **RAPID**  **n=25** | |
| --- | --- | --- | --- | --- |
|  | **Not AE**  **n=37** | **AE**  **n=11** | **Not AE**  **n=22** | **AE**  **n=3** |
| **Age at diagnosis – years** | **54(36-64)** | **54(40-62)** | **53(33-69)** | **55(39-59)** |
| **Male sex – n. %** | **28(76%)** | **9(82%)** | **19 (86%)** | **1(33%)** |
| **Smokers – n. %** | **27(73%)** | **8(73%)** | **16(73%)** | **2(67%)** |
| **Smoking history – pack-years**  **Only smokers** | **25(0.1-120)** | **28(4-70)** | **24(3-93)** | **16(5-26)** |
| **Duration of symptom before diagnosis – months** | **25(2-96)** | **47(2-75)** | **6(1-58)** | **12(4-35)** |
| **FVC at diagnosis – % predicted** | **64(20-94)*** | **43(23-69)** | **71(46-107)** | **64(58-80)** |
| **FEV1 at diagnosis - %predicted** | **66(25-97)*** | **44(26-76)** | **69(45-118)** | **65(56-78)** |
| **FEV1/FVC at diagnosis - %** | **84(71-111)** | **77(71-90)** | **80(73-89)** | **78(73-87)** |
| **DLco at diagnosis – % predicted** | **39(3-100)** | **39(15-91)** | **41 (10-74)** | **32(10-85)** |
| **FVC at end follow up – % predicted** | **54(28-87)*** | **38(21-52)** | **38(22-91)** | **38(23-40)** |
| **Follow up (from diagnosis to end study^#^)– months** | **41(7-158)** | **36(20-72)** | **24(12-60)** | **38(27-39)** |
| **6MW distance – mt** | **275(40-437)** | **260(120-400)** | **263(48-390)** | **230(160-320)** |
| **mPAP – mmHg** | **18(10-51)** | **25(10-42)** | **20(13-45)** | **23(15-30)** |
| **FVC decline/year -%predicted** | **3.2(0.0-9.1)** | **1.3(0.0-8.3)** | **14.5(10.2-28.0)** | **10.8(10.7-14.8)** |
| **FVC decline/year - ml** | **104(0-416)** | **52(0-352)** | **522(297-1498)** | **379(344-473)** |

Values are expressed as numbers and % or medians and (ranges)

**^#^** transplant, death or end follow-up

*****P<0.01 when not AE were compared to AE

**Table D. Clinical characteristics of smokers and nonsmokers**

|  | **SLOW**  **n=48** | | **RAPID**  **n=25** | |
| --- | --- | --- | --- | --- |
|  | **Smokers**  **n=35** | **Non Smokers**  **n=13** | **Smokers**  **n=18** | **Non Smokers**  **n=7** |
| **Smoking history – pack-years** | **25(0-120)** | **0(0-0)** | **24(3-93)** | **0(0-0)** |
| **Duration of symptom before diagnosis – months** | **25(2-96)** | **36(2-60)** | **9(1-58)** | **5(2-36)** |
| **FVC at diagnosis – % predicted** | **61(23-94)** | **49(20-84)** | **73(48-86)** | **58(46-107)** |
| **FEV1 at diagnosis - %predicted** | **64(26-97)** | **54(25-95)** | **71(45-99)** | **58(50-118)** |
| **FEV1/FVC at diagnosis - %** | **84(71-111)** | **83(72-90)** | **80(73-89)** | **81(50-118)** |
| **FVC at end follow up – % predicted** | **52(21-87)** | **44(25-80)** | **38 (23-69)** | **34(22-91)** |
| **AE – n. (%)** | **8(23%)** | **3(23%)** | **2(11%)** | **1(14%)** |

Values are expressed as medians and (ranges) or numbers and (%)

No differences were observed between smokers and non smokers

**Table E. Inflammatory cells in smokers and non smokers**

|  | **SLOW**  **n=27** | | **RAPID**  **n=14** | |
| --- | --- | --- | --- | --- |
|  | **Smokers**  **n=18** | **Non Smokers**  **n=9** | **Smokers**  **n=11** | **Non Smokers**  **n=3** |
| **CD45+ cells - n/mm^2^** | **348(149-653)** | **345(212-766)** | **345(212-766)** | **448(340-789)** |
| **Macrophages -n/mm^2^** | **93(27-308)** | **88(42-237)** | **134(71-340)** | **155(141-269)** |
| **Neutrophils - n/mm^2^** | **29(0.7-109)** | **18(2-95)** | **18(2-95)** | **57(3-176)** |
| **CD4+ cells - n/mm^2^** | **113(20-392)** | **150(61-564)** | **150(61-564)** | **187(113-284)** |
| **CD8+ cells - n/mm^2^** | **29(8-196)** | **41(19-156)** | **41(19-156)** | **68(44-175)** |
| **CD20+ cells - n/mm^2^** | **42(20-312)** | **42(3-389)** | **42(3-389)** | **89(51-108)** |

Values are expressed as medians and (ranges)

No differences were observed between smokers and non smokers

**Table F. Pathological characteristics in smokers and nonsmokers**

|  | **SLOW**  **n=27** | | **RAPID**  **n=14** | |
| --- | --- | --- | --- | --- |
|  | **Smokers**  **n=18** | **Non Smokers**  **n=9** | **Smokers**  **n=11** | **Non Smokers**  **n=3** |
| **Presence of DAD** | **7(39%)** | **3(33%)** | **10(91%)** | **3(100%)** |
| **Fibroblastic Foci –n/cm^2^** | **3.5(0.4-11.7)** | **3.5(1.9-10.3)** | **3.0(1.0-7.3)** | **4.6(1.1-6.1)** |
| **Lymphoid Follicles - n/cm^2^** | **6(1-16)** | **7(1-16)** | **7(2-11)** | **6(5-9)** |

Presence of DAD is expressed as number (%) of subjects with more 30% DAD in their lung sections.

Fibroblastic foci and lymphoid follicles are expressed as medians and (ranges).

No differences were observed between smokers and non smokers

**REFERENCES**

1. Boon K, Bailey NW, Yang J, et al. Molecular phenotypes distinguish patients with relatively stable from progressive idiopathic pulmonary fibrosis (IPF*). PLoS One* 2009;4:e5134.
2. Collard HR, Moore BB, Flaherty KR et al. Idiopathic Pulmonary Fibrosis Clinical Research Network Investigators. [Acute exacerbations of idiopathic pulmonary fibrosis.](http://www.ncbi.nlm.nih.gov/pubmed/17585107) A*m J Respir Crit Care Med 2007*; 176:636-43.
3. Ballarin A, Bazzan E, Zenteno RH et al. Mast cell infiltration discriminates between histopathological phenotypes of chronic obstructive pulmonary disease. *Am J Respir Crit Care Med* 2012; 186:233-9.
4. Baraldo S, Turato G, Lunardi F et al. [Immune activation in α1-antitrypsin-deficiency emphysema. Beyond the protease-antiprotease paradigm.](http://www.ncbi.nlm.nih.gov/pubmed/25412116) *Am J Respir Crit Care Med*. 2015; 191:402-9.
